# *Operando* tribochemical formation of onion-like-carbon leads to macroscale superlubricity

**DOI:** 10.1038/s41467-018-03549-6

**Published:** 2018-03-21

**Authors:** Diana Berman, Badri Narayanan, Mathew J. Cherukara, Subramanian K. R. S. Sankaranarayanan, Ali Erdemir, Alexander Zinovev, Anirudha V. Sumant

**Affiliations:** 1 Center for Nanoscale Materials, Argonne National Laboratory, 9700S. Cass Ave, Argonne, IL 60439 USA; 2 Materials Science Division, Argonne National Laboratory, 9700S. Cass Ave, Argonne, IL 60439 USA; 3 X-ray Sciences Division, Argonne National Laboratory, Argonne, IL 60439 USA; 4 Energy Systems Division, Argonne National Laboratory, 9700S. Cass Ave, Argonne, IL 60439 USA; 5 Present Address: Materials Science and Engineering Department, University of North Texas, Denton, TX 76207 USA

## Abstract

Stress-induced reactions at the sliding interface during relative movement are known to cause structural or chemical modifications in contacting materials. The nature of these modifications at the atomic level and formation of byproducts in an oil-free environment, however, remain poorly understood and pose uncertainties in predicting the tribological performance of the complete tribosystem. Here, we demonstrate that tribochemical reactions occur even in dry conditions when hydrogenated diamond-like carbon (H-DLC) surface is slid against two-dimensional (2D) molybdenum disulfide along with nanodiamonds in dry nitrogen atmosphere. Detailed experimental studies coupled with reactive molecular dynamics simulations reveal that at high contact pressures, diffusion of sulfur from the dissociated molybdenum disulfide led to amorphization of nanodiamond and subsequent transformation to onion-like carbon structures (OLCs). The in situ formation of OLCs at the sliding interface provide reduced contact area as well as incommensurate contact with respect to the H-DLC surface, thus enabling successful demonstration of superlubricity

## Abstract (web summary)

Stress-induced tribochemical reactions that reduce friction at sliding interfaces typically require liquid lubricants. Here, the authors discover the nanoscale tribocatalytic formation of onion-like carbon from 2D MoS_2_ and nanodiamond under dry and oil-free conditions, providing superlubricity at the macroscale.

## Introduction

It is estimated that nearly 1/3 of the fuel used in automobiles is spent to overcome friction, while the wear limits component life and reliability^1^. Even a modest few percent reduction in friction can significantly impact energy security, energy savings and environmental benefits. The conventional way for friction and wear reduction for most of the materials widely used in automotive industry is oil-based lubrication, but such a practice is increasingly becoming a concern mainly because of its adverse environmental impacts (oil waste) and there are efforts being made to increase the durability of oil based lubricants by introducing some additives^2,3^. Recent studies have explored tribofilm formation from zinc dithiophosphate-containing lubricant, where the growth of the tribofilm could be controlled by varying the compressive stress at the contact interface^4^. Formation of diamond-like carbon films was also observed for catalytically reacting surfaces when sliding in oil^5^. Although such studies are encouraging, the reactions often require a substantial amount of reactive additives or liquid lubricant layer in the form of oil. The viscosity of the liquid also limits the ultimate low friction that can be achieved; it is, therefore, desirable to achieve ultralow friction and wear in dry conditions as well^2^. In this context, the use of two dimensional (2D) materials such as graphene as a dry lubricant demonstrated recently is very promising^6–9^. These studies present a paradigm shift from conventional oil based lubrication and allow us to explore untapped potential of graphene and other 2D materials as a dry solid lubricant. There is a need to think about this problem in a holistic way to discover pathways for providing lubrication in an oil-free system.

Achieving superlubricity, or near-zero friction, at the macroscale sliding contact remains a major challenge^10^. In the efforts toward the structural superlubricity at macroscale, low friction has been recently observed in centimeter-long double-walled carbon nanotubes (CNT)^11^. Those experiments, however, rely on synthesis of CNTs with perfect atomic structures with long periodicity, which is very difficult to achieve for practical applications. Ultra-low friction in disordered solid interfaces such as self-mated DLC films^12–14^ and in fullerene-like nanoparticles such as MoS_2_^15^ has been observed under specific environment and sliding conditions.

In this letter, we demonstrate that in dry environment when two dimensional (2D) MoS_2_ layers are combined with nanodiamonds in an alcohol solution and drop-casted onto the SiO_2_/Si substrate surface and slid against H-DLC^16^ coated surface, the tribochemical reaction manifest formation of large OLC scrolls in situ directly at the sliding interface leading to superlubricity. Through detailed experimental investigation combined with molecular dynamics simulations, we elucidate the mechanism of OLC formation and show that a mechanical stress-induced tribochemical reaction at the nanoscale is responsible for dramatic changes at the tribological interface leading to superlubricity at the macroscale. This tribochemical reaction leading to superlubricity even in a dry environment should impact the solid lubricant field in developing long-lasting solid lubricants.

## Results

### Friction and wear studies

**Fig. 1:**
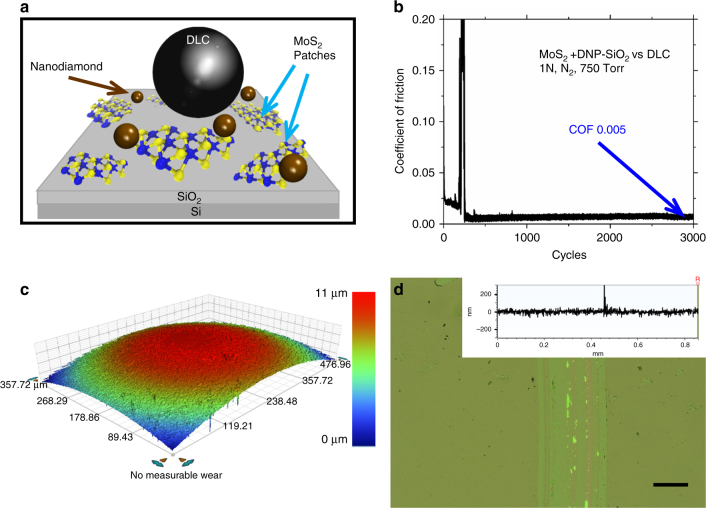
Tribological performance of MoS_2_ layers mixed with nanodiamond. **a** Schematic of the experimental setup, **b** graphs of the coefficient of friction, **c** wear of the ball, and **d** micrograph of flat sides for MoS_2_ mixed with nanodiamond and sliding against H-DLC surface. Negligible wear is indicated by line scan taken across the wear track (inset). The lowest coefficient of friction observed is 0.005 ± 0.002. The test was repeated five times with the measurement uncertainty calculated based on the variations among the tests. The scale bar in Fig. 1d is 100 μm

Figure 1 demonstrates the tribosystem tested including the initial configuration (Fig. 1a) and highlights the observed near-zero coefficient of friction (COF), reaching as low as 0.005 (Fig. 1b) with negligible wear on the ball (Fig. 1c) and flat surface (Fig. 1d). The flat mark observed on the ball side is associated with contact pressure-induced deformation (the Hertz contact diameter is approximately 86 µm) and is not due to the wear^17^. Material buildup on the flat side is associated with the formation of OLCs inside the wear track.

### The mechanism of onion like carbon formation TEM/EELS studies

**Fig. 2:**
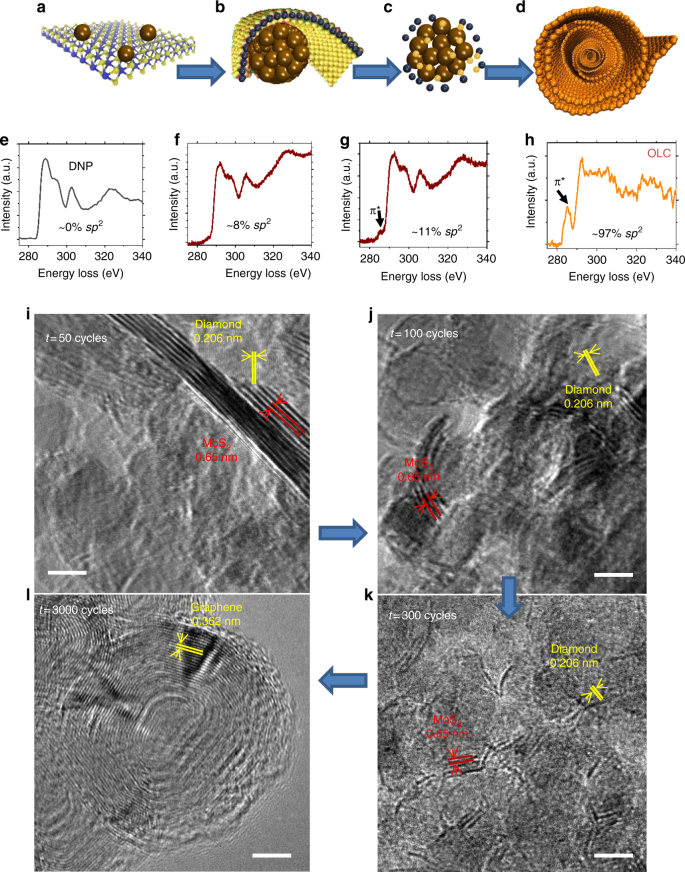
Schematics of the mechanism of the onion-like carbon formation during sliding. Schematics depicting the mechanism of OLC formation: **a** dispersion of MoS_2_ and nanodiamonds on the surface, **b** wrapping of MoS_2_ sheets around nanodiamonds, **c** partial disintegration of MoS_2_ and amorphization of nanodiamond, and **d** formation of OLCs. Electron energy loss spectra highlighting the calculated fraction of *sp*^*2*^-bonded carbon and tracking full transformation from **e** nanodiamonds into partially graphitized **f**, **g** structures and into **h** OLC structures. **i**–**l** TEM images of the wear debris taken at regular intervals after interrupting the tribotest and corresponding to four stages (**a**–**d**) of transformation. Image **i** indicates good dispersion of MoS_2_ sheets along with nanodiamonds; **j** initial breaking of MoS_2_ layers and wrapping around nanodiamonds; **k** further wrapping of them around the nanoparticles and reduction in the size of nanodiamonds due to the tribochemical reaction leading to amorphization and precipitation of amorphous carbon; and **l** complete transformation into OLCs in the wear track with no indication of MoS_2_ layers inside the wear track. The presence of the initial mixture of diamond and MoS_2_ and the OLC structures is indicated by interlayer spacing in the diamond lattice, as well as between MoS_2_ and graphitic layers. The scale bars are 5 nm

Our observations suggest a tribochemically driven mechanism of OLCs formation in the tribolayer from a mixture of MoS_2_ with nanodiamonds at sliding interfaces as shown in the schematics in Fig. 2a–d (and Supplementary Figure 1), leading to decreased friction values from high values down to near zero. The evolution of MoS_2_ and nanodiamonds leading to formation of OLC structures while sliding against H-DLC interfaces occurs via the following pathway: MoS_2_ patches with high elastic bending modulus (by a factor of 7 higher than for graphene^18^) between the sliding interfaces start to form scrolls around small clusters of nanodiamonds, as shown in Fig. 2a, b. This condition is more clear from the transmission electron microscopy (TEM) images taken on the wear debris collected from the wear track after specific wear cycles, as shown in Fig. 2i–l. As the sliding proceeds, due to the high contact pressure (~0.2 GPa), MoS_2_ starts to disintegrate into molybdenum and sulfur, and due to the high affinity of sulfur toward oxygen^19,20^, it bonds to the oxygenated nanodiamond surface. The nanodiamonds produced from this detonation process are known to have carboxyl and oxygenated species on the surface^21^. The diffusion of sulfur into the nanodiamond then takes place due to the stress-induced chemical reaction, which exhibits transformation from *sp*^3^-bonded diamond into *sp*^*2*^-bonded amorphous carbon and then eventually into graphitic layers in the form of OLCs, as shown by schematics in Fig. 2a–d and the corresponding TEM images in Fig. 2i–l (and Supplementary Figure 2). Interestingly, once disintegration of MoS_2_ occurs, amorphization and graphitization proceed immediately. Though at 300 cycles, the observed TEM image indicates beginning of the amorphization of nanodiamonds, coefficient of friction approaches low values with some variations. We attribute this effect to the fact that TEM captures only partial amount of the wear debris and certain amount of graphitization may have already started at this point.

Electron energy loss spectra (EELS) confirm this systematic evolution (with increasing *sp*^2^ fraction) in the structure of wear debris from nanodiamond (Fig. 2e–g) into OLC (Fig. 2h), which agrees with the TEM images shown in Fig. 2i–l. Initially, due to the *sp*^*3*^-bonded nature of the carbon in the detonated nanodiamonds, EELS spectra indicate almost no presence of the π^*^ peak (at ~285 eV) in the carbon K-edge spectra (Supplementary Figure 3)^22,23^. After conversion of nanodiamond into OLC structures, we observed emergence of *sp*^*2*^-bonded carbon π^*^ peak (Fig. 2g^24^ leading to fully *sp*^2^ bonded OLC structure (Fig. 2h. The corresponding *sp*^*2*^ fraction for every stage of transformation was calculated based on the position and intensity of the π^*^ peak^25^. The interaction of MoS_2_ edge atoms with the dangling bonds on the nanodiamond surface may also be helping to form a scroll around the nanodiamond, as we previously observed with a graphene-forming scroll around nanodiamond^26^. Once the nanodiamonds are fully converted into carbon nano-onions, they slide against the H-DLC surface, thus reducing the contact area and with minimal mechanical energy dissipation due to the incommensurate sliding interface between them resulting in a dramatic decrease in friction. Detailed discussion of how the graphene-scrolls-induced reduction in the contact area leads to ultralow friction values is provided in our previous work^26^.

Interestingly, dispersing the surface with only MoS_2_ flakes without the nanodiamond, but in presence of carbon-rich DLC counterpart surface did not result in the superlubricity (Supplementary Note 1). The bare MoS_2_ showed at least 10 times higher friction (COF: 0.05 ± 0.01) with high wear on the H-DLC ball side (Supplementary Figure 4). We also tried dispersing commercially available small diameter (5–7 nm) OLCs (Adámas Nanotechnologies, Inc.) directly at the interface in bare form or in combination with MoS_2_, but the OLCs did not demonstrate superlubricity and were worn out from the wear track in either case (Supplementary Figure 5). We attribute the high friction with these OLCs to their being only a few layers thick (5–7 layers), as shown in Supplementary Figure 5a, as compared to the in situ produced multilayers (15–20 layers) of OLCs in the previous case (Fig. 2l). We believe that at such low layer thickness, the overall stiffness of the OLC is not high enough to withstand high contact pressures and shear stresses at the interface. As a result, they buckle during sliding, increasing the contact areas and hence displaying relatively higher friction. By contrast, the multilayered OLCs survive high contact pressure due to their higher stiffness. On the basis of molecular dynamics (MD) simulations, we explain this subtle relationship in the stiffness with respect to the number of graphitic layers in later part of this manuscript.

### Raman spectroscopy and LDSPI studies

To gain further insight into the evolution of the carbon-based tribolayer within the wear track and identify the chemical state of the MoS_2_, we have carried out Raman spectroscopy studies and time-of-flight laser desorption single photon ionization (LDSPI) analysis of the wear track. The LDSPI analysis is a very sensitive technique, detecting elemental compositions down to the parts per billion (ppb) level without the risk of modifying the surface (such as alloying) as it employs gentle laser desorption as opposed to the ion-beam induced sputtering used traditionally with time-of-flight secondary ion mass spectroscopy (ToF-SIMS). More details about this technique and specific advantages are mentioned elsewhere^27^.

**Fig. 3:**
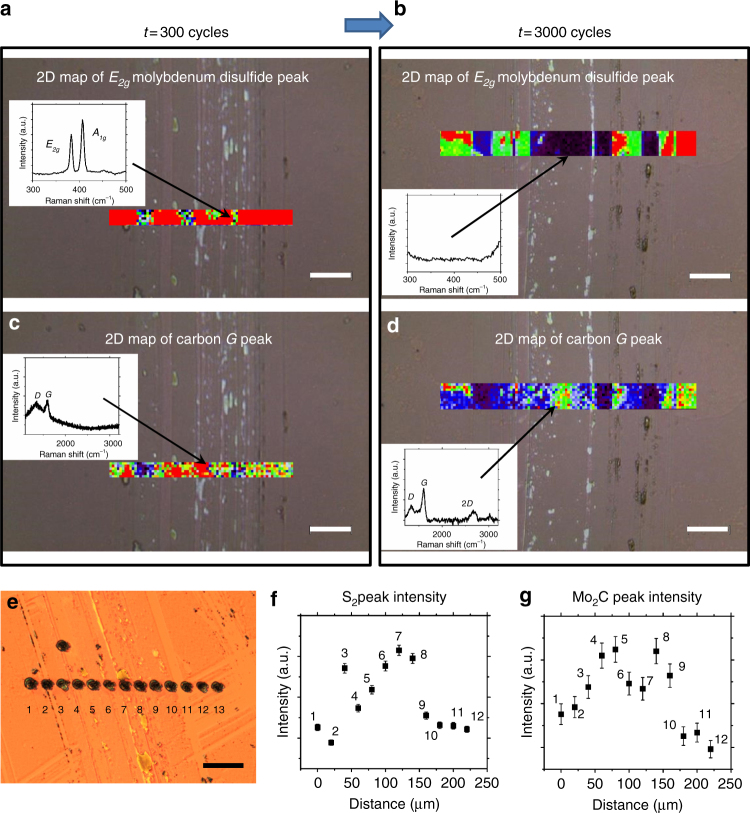
Surface analysis of the wear track. Raman 2D mapping of the wear track for the characteristic *E*_*2g*_ peak (at ~383 cm^−1^) of molybdenum disulfide and characteristic *G* peak (at ~1600 cm^−1^) of carbon-layered structures after initial 300 cycles (**a**, **c**) and 3000 cycles (**b**, **d**). Insets demonstrate typical MoS_2_ (**a**, **b**) and graphitic carbon (**c**, **d**) signatures inside the wear track. **e** Twelve points across the wear track for which LDSPI analysis performed. Increase in intensity for **f** molecular sulfur S_2_ peak at 64 atomic mass units and **g** molybdenum carbide Mo_2_C peaks at 204 atomic mass units indicates transformation of molybdenum and sulfur after disintegration of molybdenum disulfide. The samples were analyzed immediately after the test without cleaning. The scale bars are 50 μm

As shown in Fig. 3a, c, the Raman 2D mapping of the characteristic *E*_*2g*_ peak for MoS_2_ (at ~383 cm^−1^) and the *G* peak for carbon structures (at ~1600 cm^−1^) after initial 300 wear cycles indicates uniform distributions of carbon and MoS_2_ patches inside the wear track similar to the ones on the unworn surface outside the wear track. However, after 3000 cycles, when TEM analysis shows no traces of MoS_2_ in the form of two-dimensional layers, Raman mapping of the *E*_*2g*_ characteristic MoS_2_ peak shows no MoS_2_ in the center of the wear track, and Raman mapping shows increased intensity of the carbon G peak at the center of the wear track compared to that of the unworn surface (Fig. 3b, d). This higher G peak indicates formation of *sp*^*2*^-bonded carbon in the wear track, as one would expect from the graphitization of the *sp*^*3*^-dominated nanodiamond structure.

The LDSPI analysis was performed at twelve points across the wear track (see the optical micrograph in Fig. 3e). The resulting data further support the observation that MoS_2_ disintegrates during the sliding test possibly under high shear and high mechanical stress conditions as a consequence of subtle changes in the molybdenum, sulfur, and carbon compositions (Fig. 3e–g). No sulfur atom signal (at 32 atomic mass unit, amu) was detected across the wear track, whereas a signal at mass 64 amu, attributable to the sulfur molecule S_2_, was clearly observed (Supplementary Figure 6). This result is not surprising because of the high ionization potential and low ionization probability of S in comparison with S_2_^28^. Figure 3f, g illustrate the variation of the S_2_ peak and molybdenum carbide (Mo_2_C) signals, respectively, when analysis spots are rastered point-by-point across the wear track. The S_2_ and Mo_2_C signals increase inside the wear track in comparison with the outside area, suggesting the formation of free sulfur (presumably in the form of S_2_) and molybdenum carbide molecules during the wear test. Note that although with LDSPI we see increased S_2_ and Mo_2_C signals within the wear track, it is quite insignificant at the TEM scale (unless we use aberration-corrected high resolution TEM). In order to further investigate disintegration of MoS_2_ within the wear track and determine the final fate of disintegrated elements, we have employed combination of electron and X-ray analysis techniques (Supplementary Figures 7–9, Supplementary Note 2). The Raman data on the global scale, TEM/EELS data on the local scale, and additional key information regarding the elemental and chemical species using combination of AES, XPS, and LDSPI data bring out the key features of the chemical evolution of the tribolayer taking place and strengthen our argument regarding the mechanism of OLC formation within the wear track.

### Reactive molecular dynamics simulations studies

To better understand the tribochemical mechanism that leads to such a dramatic friction reduction, we performed large-scale reactive molecular dynamics (RMD) simulations. Vasu et al.^29^ reported that van der Waals forces create high contact pressure on the order of gigapascals on the molecules trapped between 2D layers. Meanwhile, Ashby et al.^30^ showed that during dry sliding, the local contact heating events may result in a temperature increase up to 1773 K. Thus, under the experimental conditions employed in the present study, it is quite reasonable to assume that once MoS_2_ wraps around nanodiamond clusters, the high contact pressure (1–2 GPa) and strong van der Waals forces acting on these trapped nanodiamonds can lead to slow disintegration of MoS_2_ into its constituent elements (i.e., Mo and S); subsequently, Mo and S atoms can interact with the nanodiamond in the wear track. We investigated disintegration of MoS_2_ at high contact pressure via RMD simulations by applying constant load on few layer MoS_2_ sheets (Supplementary Figure 10). The high contact pressures (~1 GPa) on MoS_2_ are indeed seen to cause significant structural disorder as seen from these simulation results suggesting it is very likely that MoS_2_ can rupture in a physical sense (Supplementary Figure 11). Interestingly, we observed that rupture of few layers of MoS_2_ layers is easier than single layer of MoS_2_. Additionally, it is also likely that the detonated nanodiamonds have complex surface chemistry^22^ involving  oxygenated species and dangling carbon bonds on the surface. We believe that apart from high contact pressures caused by wrapping of MoS_2_ around nanodiamond, the presence of oxygen and dangling bonds on the nanodiamond surface may provide reactive sites for the MoS_2_ to chemically react with nanodiamond, and thereby, facilitate its disintegration. It is important to note that during the initial run-in period of a few 100 cycles (Fig. 1b), the friction is very high, which might raise  the local temperature, and in turn, accelerate this tribochemical degradation process. Modeling such complex chemistries are beyond the reach of classical MD potentials currently available and would represent a very interesting future study.

**Fig. 4:**
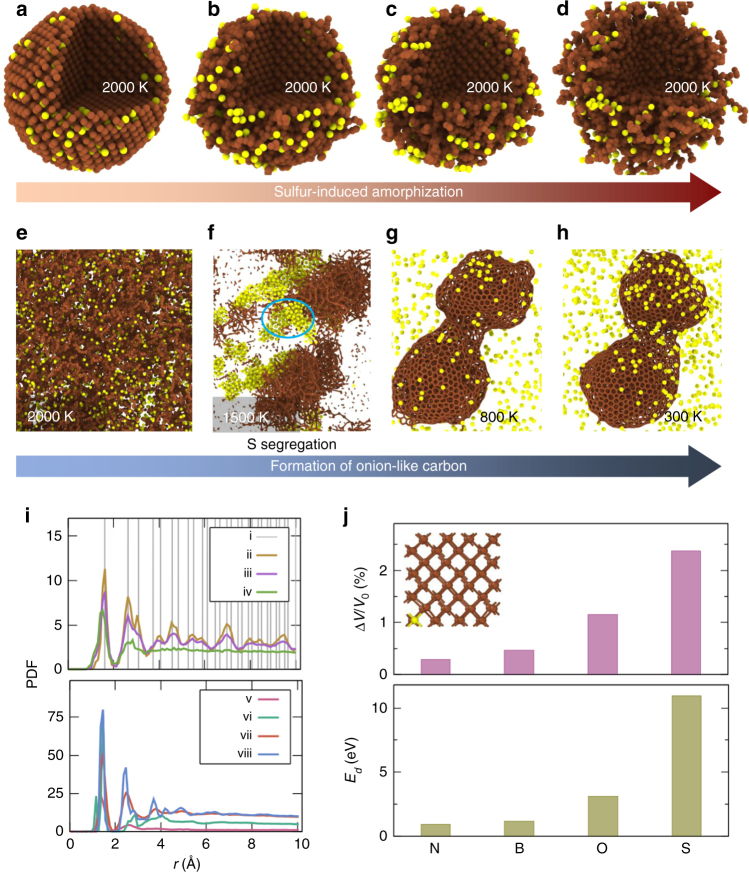
Atomistic simulations of the tribochemical mechanism leading to reduction in friction. **a**–**d** Atomic snapshots from RMD simulations at selected times during the sulfur-induced amorphization of diamond nanoparticle. **e**–**h** Atomic snapshots from RMD simulations at selected times during the formation of OLC structures from amorphous carbon matrix containing uniformly dispersed S atoms (15% S concentration). **i** pair distribution functions of C-C for each snapshot shown in **a**, **b**. **j** Volumetric strains in the diamond lattice (Top) induced by substituting a C atom with different impurity atoms, and the corresponding defect energies *E*_d_ (Bottom) obtained from DFT calculations. In panels **a**–**d**, and **e**–**h**, the temperatures corresponding to the atomic snapshots are provided. In panel **j**, Δ*V* refers to change in the volume of diamond lattice owing to introduction of a substitutional defect, and *V*_0_ refers to the volume of diamond lattice at equilibrium

We performed atomistic simulations to gain insights into the interaction of Mo and S atoms with nanodiamonds (Fig. 4). Our RMD simulations indicate that both S and Mo induce structural degradation of the nanodiamonds: S induces rapid amorphization of the diamond lattice as shown in the snapshots in Fig. 4a–d, whereas Mo reacts locally with the neighboring C (Supplementary Figure 12) to form molybdenum carbide (as also confirmed experimentally), which is thermodynamically feasible at the high temperatures (>1500 K) observed under dry sliding conditions^31^. In the case of S, the large steric size of the S impurity causes strong S–S interaction, which is mediated by the distortion of the diamond lattice up to the next nearest-neighbor lattice sites. Our simulations at varying S content of 1–15% suggest that amorphization occurs at the percolation threshold of the S–S network with the next nearest-neighbor connectivity (~10% S concentration). For example, the snapshots in Fig. 4a–d follow the structural change in the diamond lattice upon introducing S impurities at a concentration of 15% at 2000 K, which is representative of the asperity-level flash temperatures typically achieved during dry sliding^30^. The larger S atoms result in large localized strains (near the impurity site), which propagate through the diamond lattice and cause significant lattice distortion and disordering. The structural disorder manifests itself as a broadening of nearest-neighbor peaks, as well as progressive disappearance of higher order peaks in the C–C pair distribution functions (PDF) (Fig. 4i). The final structure is thus highly disordered, comparable to that of amorphous carbon (Fig. 4d). This S-induced disordering of the diamond lattice is observed regardless of the initial spatial distribution of the impurity S atoms. In a representative case, when all S atoms are initially placed only on the surface of nanodiamond, the S atoms diffuse into the sub-surface layers of nanodiamond, and induce large local strains. Subsequently, as aforementioned, the localized strains propagate through the lattice, causing disordering, i.e., amorphization of the nanodiamond (Supplementary Figure 13).

Next, we tracked the structural evolution of this amorphized carbon matrix containing 15 at% S impurities (obtained via S-induced disordering of diamond nanoparticle, Fig. 4a–d) upon cooling from 2000 to 300 K over a period of 2 ns (Fig. 4e–h) using RMD simulations. During the first ~0.5 ns of this cooling run, we observed significant segregation of S atoms, resulting in C-rich regions. Thereafter, graphitic carbon rings nucleate within the C-rich regions over the next ~0.2 ns. These nuclei, then, grow progressively into carbon nano-structures over the remainder of the run (i.e., ~1.3 ns); atomic re-arrangements occur within these nanostructures resulting in a highly ordered arrangement (Fig. 4h). The increase in C-C ordering in the formed carbon nanostructures is evidenced by the appearance of sharp peaks at characteristic separation distances in the C-C PDFs (Fig. 4i) Interestingly, the C-C PDFs reveal a prominent shoulder appearing at ∼2.85 Å during the crystallization stage, the intensity of which increases with time (Fig. 4i). This peak corresponds to intra-hexagon spacing in graphite, which suggests the formation of a more compact, homogeneous, and less defective structure with graphitic order. Analysis of the crystallized structure reveals an onion-like texture, which consists of numerous graphitic hollow spheres arranged concentrically in a layer-by-layer manner as confirmed experimentally by our TEM observations. Note that the final OLC structures obtained from our RMD simulations contain trace amounts of embedded S atoms (<1%); the strain induced by trace amounts of S is not sufficient to perturb the crystallinity (i.e., graphitic order) of OLC. The formation of these OLC structures has interesting ramifications for subsequent tribological processes.

To elucidate the load-bearing properties of the shell structures, we characterized their response to compressive uniaxial stress through MD simulations (Supplementary Figure 10). Here we should highlight that the initial high stiffness of the MoS_2_ layers allows for encapsulation of large clusters of nanodiamonds into MoS_2_ shells. Such shells play the role of a template for the formation of OLC structures of large size. The experimental results indicate that the typical diameter of the scrolled structures is in the range of 20–30 nm (and up to 40 layers). The atomistic snapshots shown in Supplementary Figure 10 for a representative onion configuration with 5 layers, in which atoms are colored on a scale related to their potential energy, depict the structural evolution under an applied load. As shown in this simulation, the dimensional reduction along the compression direction is accompanied by significant volume expansion along the axial directions.

We quantified the maximum sustainable load (MSL) for a given number of layers in a carbon onion (Fig. 4i). With increasing number of layers, the MSL is also expected to increase (e.g., a linear extrapolation estimates MSL for 40-layer onion to be ~3.5 µN) which is experimentally shown to be capable of supporting significantly higher loads without failure. Our simulations and experiments suggest that a stable superlubricity regime is possible with OLC diameters in the range of 20–30 nm.

The capability of layered structures to carry high loads and provide low friction has been previously shown for sputtered MoS_2_ films^15^.

In addition to probing the tribochemical origin of the drastic reduction in friction, we also employed density functional theory calculations to investigate the possibility of achieving amorphization of diamond (and subsequent crystallization into OLC) via other elements. We estimated the ease of substituting a carbon atom in the diamond lattice with different impurity atoms (namely, N, B, O, and S) by computing the corresponding defect energies (*E*_d_) using DFT calculations (Methods). Note that, any of these impurity atoms are unlikely to be incorporated in interstitial voids in diamond lattice, since they are highly unfavorable energetically (Supplementary Table 1). Our DFT calculations show that atoms with small radii (i.e., B and N) can substitute C atoms in diamond lattice with low energy penalty (*E*_d_ < 1.2 eV); O and S defects are associated with higher energies. Nevertheless, such defect energies can be surmounted under the extreme conditions afforded by tribology at nanoscale. Although substitutional defects of each of these impurities can occur at tribological interfaces, B, N, and O atoms induce low local strains (volumetric strain <0.5%), which is unlikely to cause significant distortion/disordering of the diamond lattice (Fig. 4j). The slightly larger S atom (1.15 Å) enables much higher local strains (~2.37% volumetric strains) in the diamond lattice, and thereby, facilitates amorphization of nanodiamond.

## Discussion

The picture that emerges from our experimental and theoretical investigations is that, after initial encapsulation of nanodiamond clusters by 2D MoS_2_ layers, the high contact pressure during sliding causes gradual disintegration of MoS_2_ layers. We find that S atoms from the surface diffuse into the sub-surface layers of nanodiamond sphere. The diffusion of sulfur into nanodiamond possibly takes place via interactions of sulfur with the oxygenated surface of the nanodiamond and induces large local strain causing significant lattice distortion. The mechanical stress-induced tribochemical reaction eventually induces graphitization of *sp*^*3*^-bonded carbon all the way to the core of the cluster (Fig. 4a–d) and initiates formation of concentric OLC structures (Fig. 4e–h). As suggested by Xie et al.^32^, sulfur plays an important role in graphene formation starting from dehydrogenation of the outer shell of the diamond nanoparticles and followed by formation of bridging monosulfide linkages, which are further rearranged into the graphene lattice. Molybdenum also amorphizes nanodiamonds^33,34^; however, the graphitization rates are lower than those for sulfur (Supplementary Figure 10), and during the metal-induced catalytic graphitization, the molybdenum forms stable molybdenum carbide compounds (Fig. 3g), which preclude onion formation. The in situ formation of OLCs reduces the effective contact area and provides an incommensurate surface against randomly oriented DLC^35^, thus reducing the friction coefficient to the superlubric regime by a similar mechanism as for graphene scrolls described in our earlier work^26^ with the exception that no tribochemical effect was observed in earlier case protecting the nanodiamond from disintegration.

To summarize, we have discovered a stress-induced tribochemical mechanism proceeding in dry atmospheric conditions leading to macroscale superlubricity. The tribochemical reaction of sulfur with nanodiamond results in pressure- and stress-induced transformation of nanodiamond clusters into in situ formation of *sp*^*2*^ bonded graphitic layers arranged into OLC structures, which are capable of providing the superlubricity when sliding against an amorphous H-DLC surface. This discovery will have impact in developing oil-free solid lubricants for automotive and related applications.

## Methods

### Preparation of materials

Solution-processed molybdenum disulfide was prepared by chemical exfoliation of bulk MoS_2_ crystal and was then suspended in ethanol with 18 mg L^−1^ graphene. The resulting solution contained 1–8 monolayers thick MoS_2_ flakes. Next, we added diamond nanoparticles (nanodiamonds) of 3–5 nm diameter (Supplementary Figure 3) into the solution in the proportion of 50–1000 mg of nanodiamonds per liter of solution. The resulting solution after 20 min of sonication was deposited in a small amount (10–20 drops or 0.5–1 mL of solution per 10 cm^2^) on the SiO_2_ substrate in a colloidal liquid state, and the liquid ethanol was evaporated in dry nitrogen. This procedure resulted in few-layer-thick MoS_2_ flakes (~75% of the surface coverage with the estimated flakes size of 0.2–0.5 µm) and nanodiamonds non-uniformly covering the substrate. The expected number density of nanodiamonds per unit area of the substrate is in the range of 10^11^–10^13^ particles per cm^2^, depending on the size of the nanodiamonds (3–5 nm). Commercially available OLCs (Adámas Nanotechnologies, Inc.) have been deposited on the silicon surface from the OLC-containing ethanol solution.

In the ball-on-disk tests described below, the counterpart was a stainless steel ball (440C grade) of 9.5-mm diameter covered with a 1-µm-thick hydrogenated diamond-like carbon (H-DLC) layer of root mean square roughness *R*_q_ = 20 nm. The DLC film was deposited by plasma-enhanced chemical vapor deposition at room temperature^12^.

### Tribological tests

Tribological tests were performed in dry nitrogen (900 mbar) and humid air (30% relative humidity) at room temperature using a CSM ball-on-disk macroscale tribometer. The normal load during the tribotests was kept at 1 N (Hertz contact pressure of 0.2 GPa), and the angular velocity was 60 rpm (0.6–9 cm s^−1^ where the radius of the wear track varied from 1 up to 15 mm). Zero calibration of the machine was performed automatically at the beginning of each test. All the tests were repeated at least five times to confirm reproducibility of the results. The error bars are calculated based on the variations between the tests.

The wear volume of the flat was very difficult to assess, as wear was manifested as deep scratches and could not be fit into a reliable wear equation. To estimate the wear volume for the balls after the tribotests, we used the following equation:

1 $$V = \left( {\frac{{\pi h}}{6}} \right)\left( {\frac{{3d^2}}{4} + h^2} \right)$$

where

2 $$h = r - \sqrt {r^2 - \frac{{d^2}}{4}}$$

*d* is wear scar diameter, and *r* is the radius of the ball.

### Characterization techniques

The wear scars were imaged with an Olympus UC30 microscope and characterized by an Invia Confocal Raman microscope using the red laser light (*λ* = 514 nm). The wear debris formed during the tribotests was imaged with a JEOL JEM-2100F transmission electron microscope, for which samples were picked up from the wear track with a probe and transferred to a copper grid. Laser desorption analysis was performed with a home-built, time-of-flight mass spectroscopy SARISA (surface analysis by resonant ionization of sputtered atoms) instrument^36^.

Laser desorption single photon ionization analysis was performed with a laser post-ionization secondary neutral mass spectrometry instrument operated in the laser desorption mode^36^ using the second harmonic of Ti:sapphire (370 nm wavelength, 14 ns pulse duration). The desorption laser pulses were focused onto the front side of the target, with the use of an instrument-embedded microscope, into a spot of about 7-micron diameter. An F_2_ laser (GAM 100EXF, *λ* = 157 nm, 10 ns pulse duration, 2 mJ per pulse energy) was used to photoionize the desorbed species in the plume with the delay of 2000 ns against the desorption laser pulse. Ionized atoms and molecules were collected by front optics and analyzed by a time-of-flight mass spectrometer. The instrument was operated with 200 Hz repetition rate. The target was positioned on the in situ nano-motion stage, and the analysis was performed at several points by moving the target across the desorption laser beam in increments of 20 µm. Each mass spectrum was the sum of 1024 laser shots acquired by a fast digitizer. Considering the Poisson  statistics of the signal, the error bars in Fig. 3 were calculated as the square root of the signal.  Each point on Supplementary Figure 6 is the result of integration of appropriate mass signal in the spectrum over the mass in the range *M*_max_ ± Δ*M*, where *M*_max_ is the mass that corresponds to the peak maximum, and Δ*M* denotes the signal drop to 10% of its maximum.

### Auger and X-ray photoelectron spectroscopy

Auger spectroscopy analysis has been performed by a PE/PHI (Perkin-Elmer) model SAM 660 system with a single pass cylindrical mirror analyzer. For in-depth elemental analysis of the wear track the samples were sputtered with a differentially pumped 1–5 keV argon ion gun every for 12 second periods of time and the following Auger spectrum has been acquired.

X-ray photoelectron spectroscopy analysis (XPS) has been performed with a PE/PHI model 5400 XPS system equipped with hemispherical energy analyzer operated at a pass energy of 17.9 eV. The system used Mg *Kα* radiation (400 W, 15 kV).

### Reactive molecular dynamics simulations

The interactions between C, Mo, and S atoms was determined by using a reactive force field (ReaxFF) based on bond order formalism. The ReaxFF parameters employed were taken from Mattsson et al.^37^. ReaxFF provides a continuous treatment of formation/dissociation of bonds and dynamic charge transfer between atoms, and thereby, it describes chemical reaction pathways accurately. To understand the impact of S and Mo atoms on the structure of diamond nanoparticles, we input that the surface of the nanodiamond was doped with Mo/S atoms at random locations (concentration ranging from 1 to 15%). We employed nanodiamonds ~3 nm in diameter (7200 C atoms) for all the simulations. All the atoms were imparted with velocities of Maxwell distribution, such that their kinetic energy was consistent with 2000 K; structural evolution of the nanodiamond containing S impurities was then monitored in canonical RMD simulations with a time step of 0.25 fs in LAMMPS^38^; the constant temperature conditions are maintained using Nosé-Hoover thermostat^37^. First, the impact of S-impurities on the diamond lattice is investigated at 2000 K for 1 ns; thereafter, the system is cooled from 2000 to 300 K over 2 ns. To simulate the size effect on the load-bearing properties of the onion shell structures, we generated onion structures with 3, 4, 5, and 7 layers and studied their response to compressive uniaxial load (up to micro-newton range) through MD simulations. The fracture load was identified by the sudden discontinuity or drop in the load vs. displacement curve.

### Density functional theory calculations

All the density functional theory calculations were performed within the generalized gradient approximation in the projector augmented plane wave formalism implemented in VASP^39,40^. The exchange correlation is described by the Perdew–Burke–Ernzerhof (PBE) functional^41^ using the pseudopotentials supplied by VASP. To calculate the defect energy associated with substituting a C atom in diamond lattice with various impurities (B, S, Se, Te), we employed a computational supercell consisting of 8 unit cells of diamond (64 atoms). An arbitrarily chosen C atom is then replaced with the given impurity atom to create the defective configuration. Periodic boundary conditions are employed along all directions. The plane-wave cut off is set at 520 eV; we employed a Γ-centered 6×6×6 k grid to sample the Brillouin zone. The atomic positions, as well as the supercell volume and shape are optimized until the total energy converges to within 1 meV per atom. The substitution energy *E*_d_ is defined as *E*_d_ = *E*_def_ *− N*_c_ *E*_c_ − *N*_*i*_*E*_*i*_, where *E*_def_ is the total energy of defective configuration, *E*_c_ is total energy (per C atom) of pristine diamond lattice, and *E*_*i*_ is the total energy of an impurity atom in its reference state, while *N*_c_ and *N*_*i*_ are number of C and impurity *i* atoms, respectively. For B, and S, the most stable crystalline polymorph is chosen as the reference state, while for O and N the gaseous molecules O_2_ and N_2_ are chosen as reference states respectively. We employed the correction factors derived from recent high-throughput DFT calculations^42^ to alleviate the well-known errors of GGA-PBE in describing energetics of molecular species O_2_ and N_2_.

### Data availability

The authors declare that the data supporting the findings of this study are available within the article and its supplementary information files.

## Electronic supplementary material

Supplementary Information(PDF 1358 kb)

Description of Additional Supplementary Information(PDF 50 kb)

Supplementary Movie 1
