# Supplementary material for: Operando tribochemical formation of onion-like-carbon leads to macroscale superlubricity
Source: Nat Commun. 2018 Mar 21;9:1164. doi: 10.1038/s41467-018-03549-6 (PMC5862981; doi:10.1038/s41467-018-03549-6)
Supplement: Supplementary file 1 — Supplementary Information(PDF 1358 kb) [file 41467_2018_3549_MOESM1_ESM.pdf]

Supplementary Materials

Operando Tribochemical Formation of Onion-Like-Carbon Leads to Macroscale Superlubricity

Berman et al.

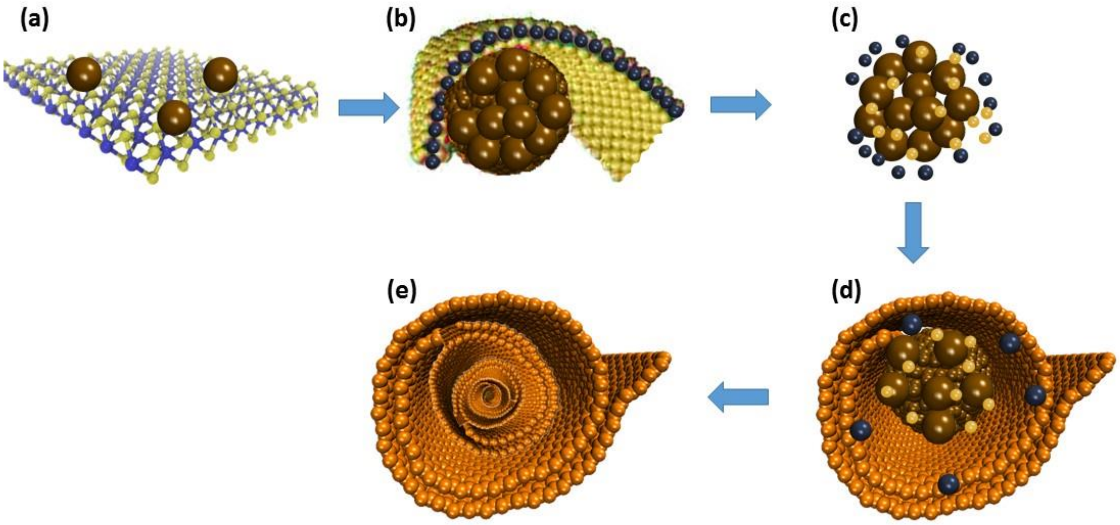

Supplementary Figure 1. Suggested mechanism for OLC formation inside the wear track.

Schematic of the catalytically induced transformation of (a) MoS_2 + nanodiamond mixture toward OLC formation: during initial sliding (b), MoS_2 wraps around the clusters of diamond; and under applied contact pressure and local heating, the following (c) disintegration of MoS_2 and (d) graphitization of nanodiamonds occurs toward (e) eventual formation of the OLC. Blue color indicates atoms of molybdenum, yellow - atoms of sulfur, brown – sp^3-bonded nanodiamond structures, and orange – sp^2-bonded carbon layers.

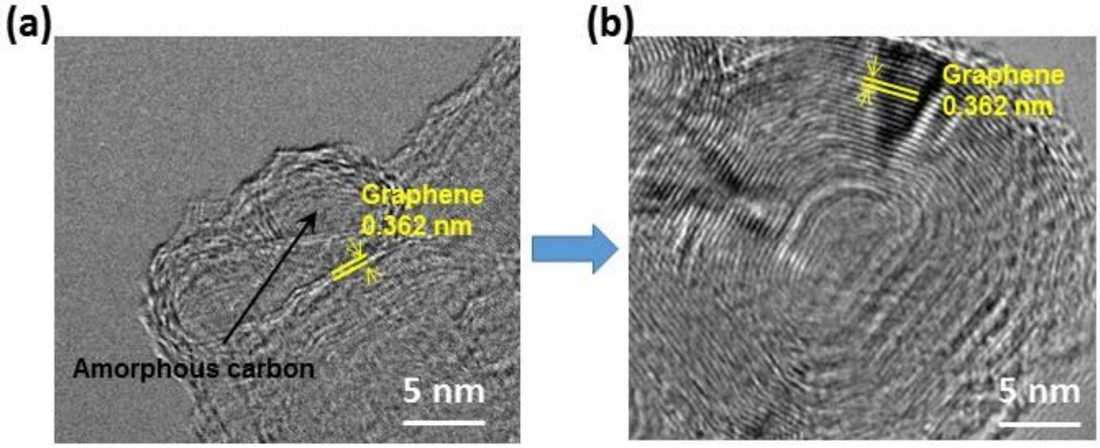

Supplementary Figure 2. Formation of OLC from graphitized carbon.
TEM observation of the wear debris indicates (a) partial graphitization of the scroll shell with the core being amorphized, followed by eventual formation of (b) a fully ordered OLC structure. The intermediate state in transformation when diamond is partially graphitized is very difficult to observe because, once disintegration of MoS_2 occurs, the following graphitization proceeds immediately.

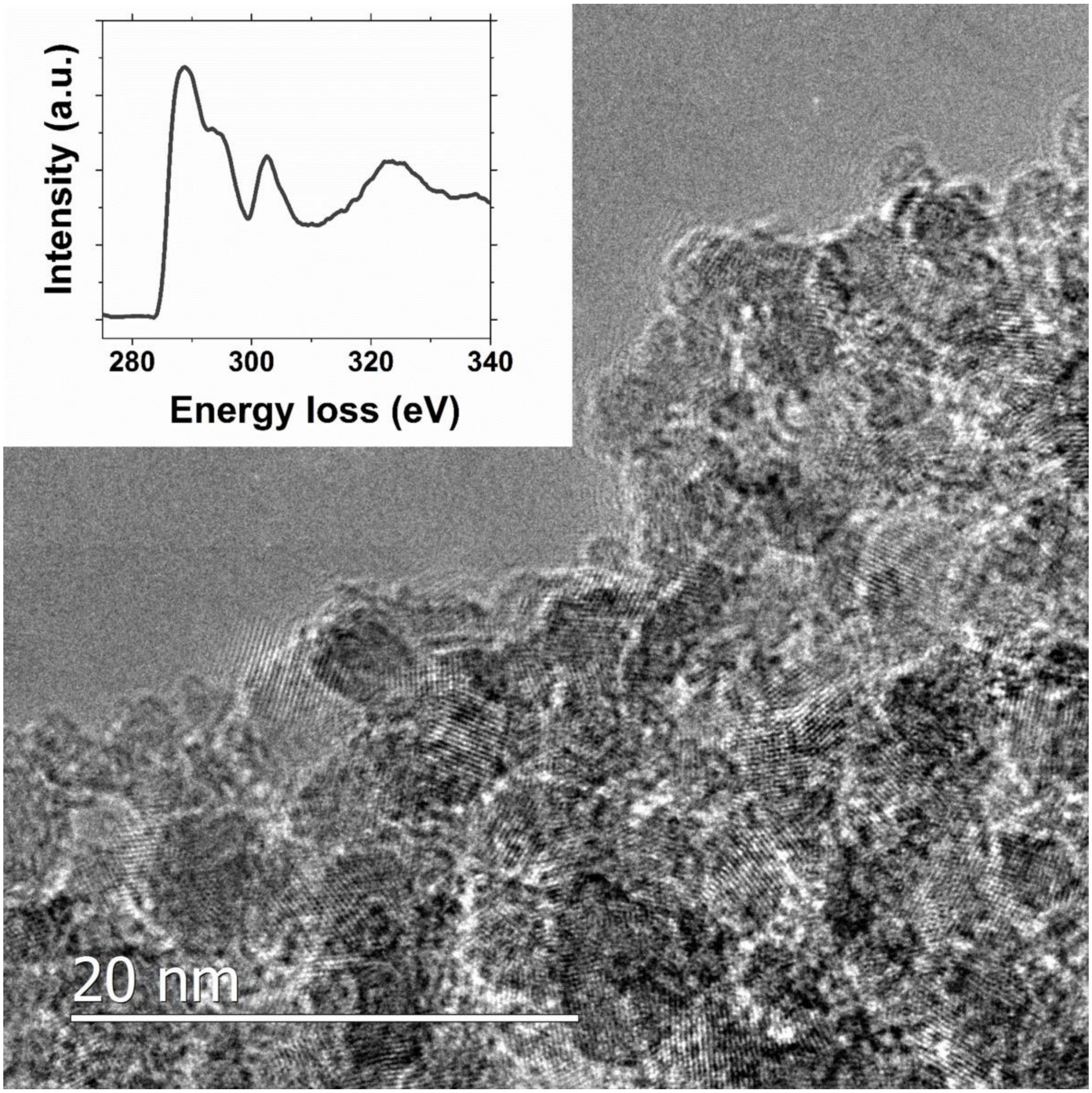

Supplementary Figure 3. Diamond nanoparticles from solution.
TEM image of nanodiamonds before the tribology tests with inset indicating typical EELS signature of nanodiamond, consisting predominantly *sp*^{3} bonded carbon without any grahitic layers.

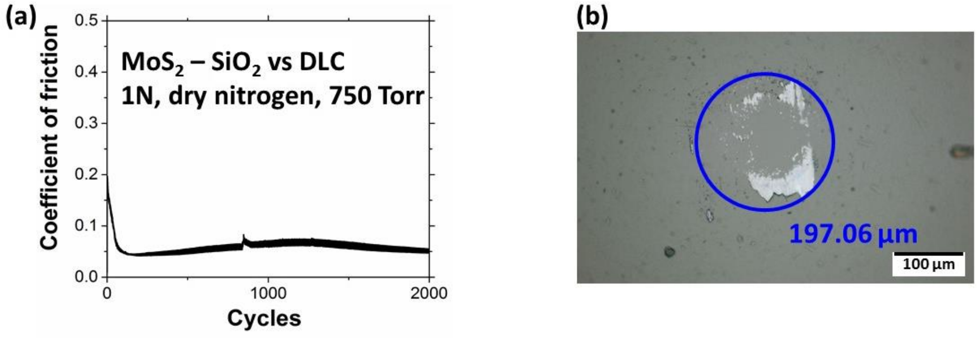

Supplementary Figure 4. Coefficient of friction (COF) and wear of bare MoS_2 of silicon dioxide.

(a) The COF for MoS_2 on silicon dioxide substrate sliding against DLC ball in dry nitrogen conditions and (b) the wear of the ball side after the test. The COF observed for molybdenum disulfide sliding against DLC ball without nanodiamonds is 0.05 +/- 0.01. The wear volume observed equals 9.25 +/- 1.26 x 10^-6 mm^3.

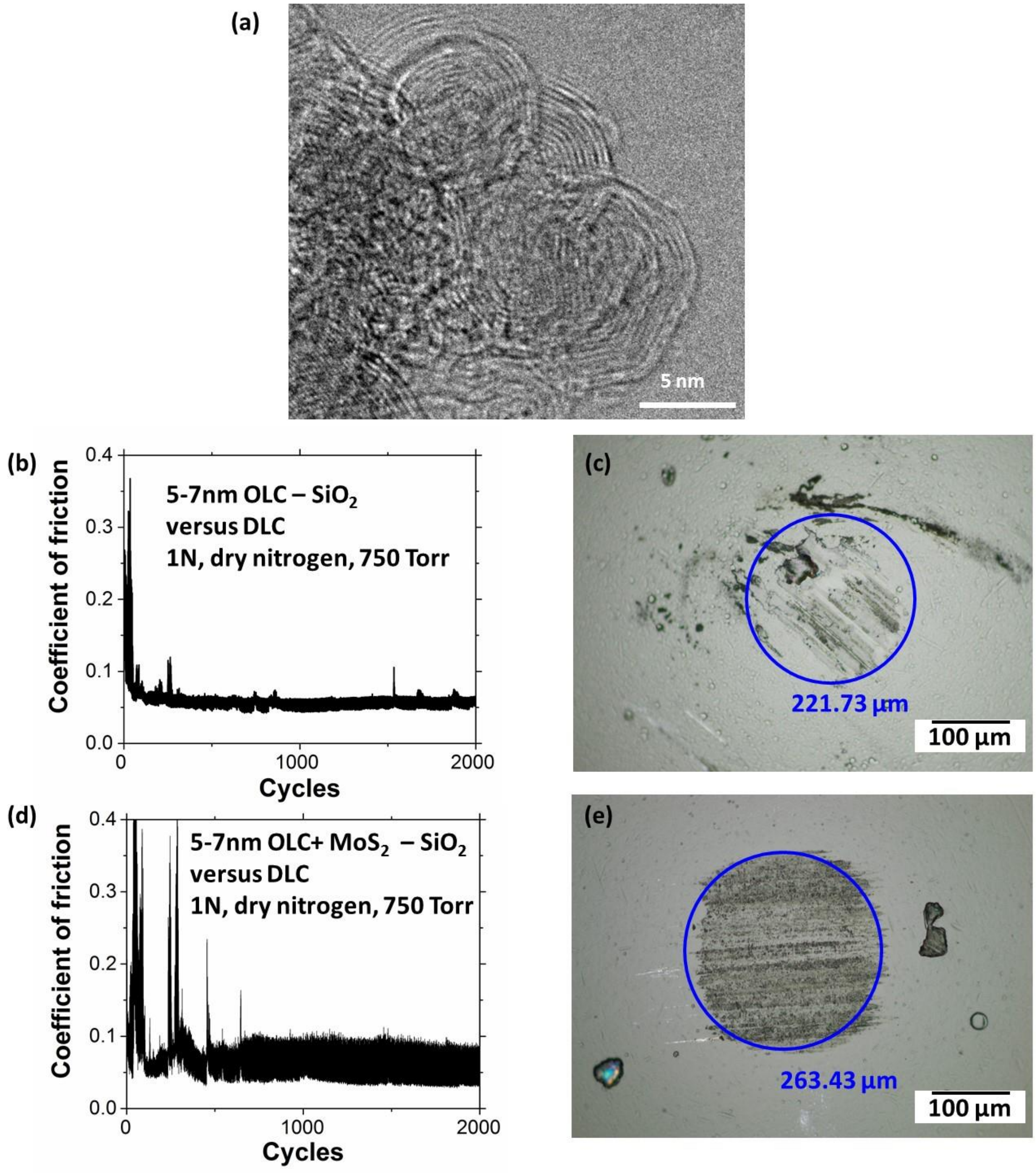

Supplementary Figure 5. Tribological performance of small manually supplied OLCs.

(a) TEM image demonstrates the structure and the size of the OLCs deposited on the surface. (b) COF for 5-7 nm OLC on silicon dioxide substrate sliding against DLC ball in dry nitrogen indicates high frictional values: 0.06 +/- 0.012. (c) The wear of the ball side after the tribo-test indicates a wear volume of 1.48 +/-0.28 x 10^-5 mm^3. (d) COF for 5-7 nm OLC combined with molybdenum disulfide flakes on silicon dioxide substrate sliding against DLC ball in dry nitrogen indicates high frictional values: 0.05 +/- 0.02. (e) The wear of the ball side after the tribo-test indicates wear volume of 2.96 +/- 0.47 x 10^-5 mm^3.

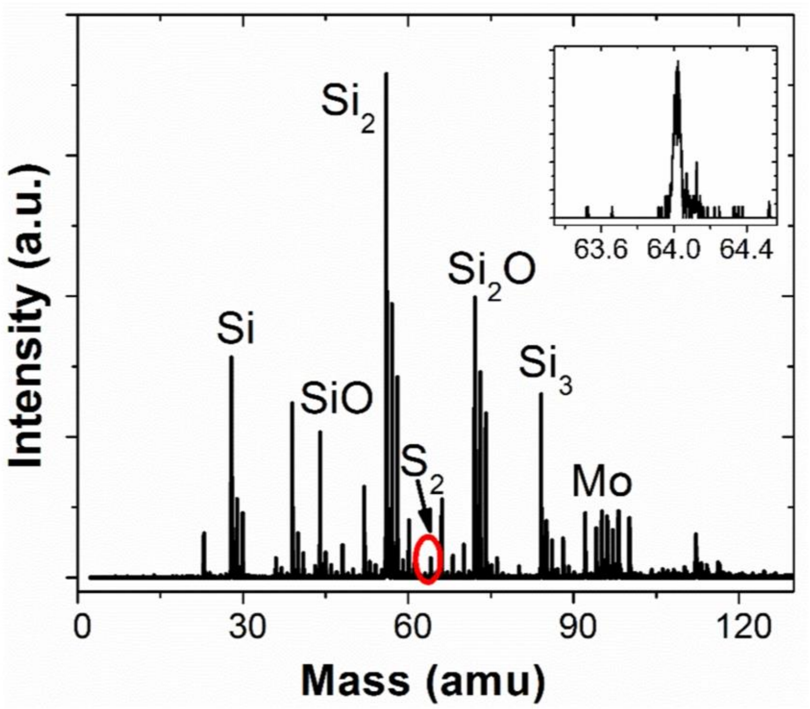

Supplementary Figure 6. LDSPI analysis of the wear track.
Typical survey scan highlighting position of sulfur peak, with inset indicating detailed scan for sulfur.

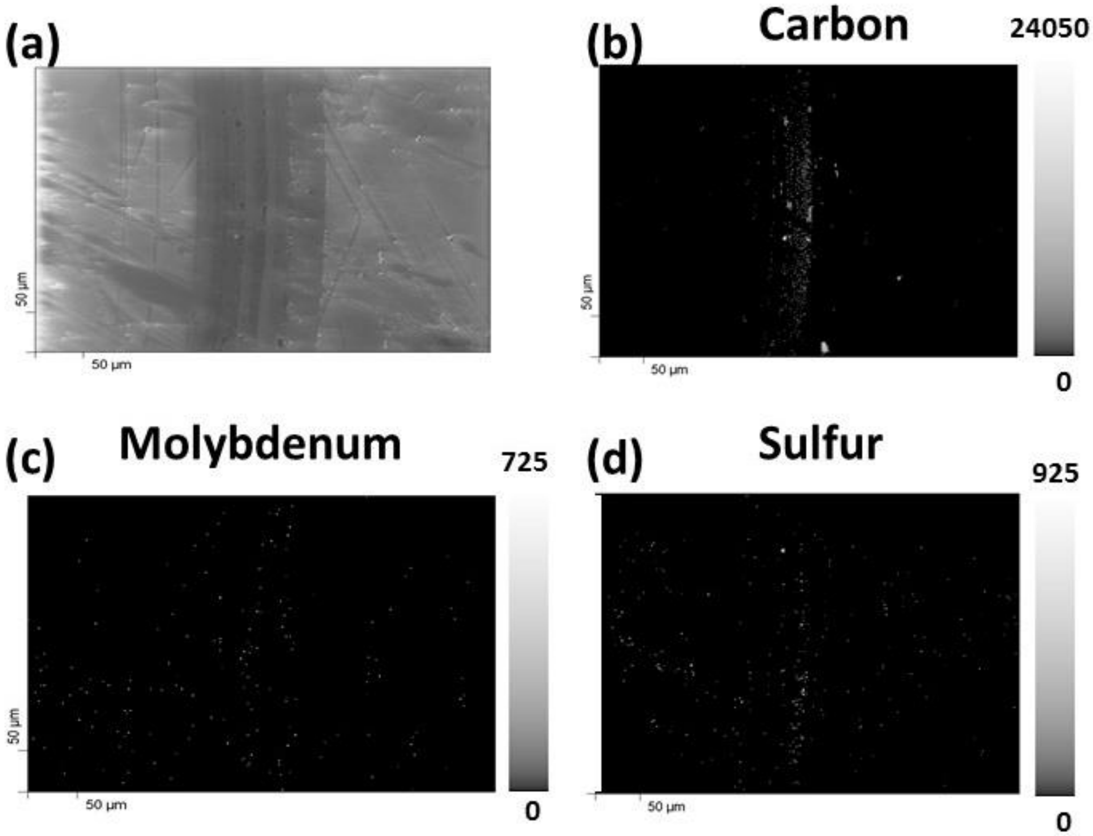

Supplementary Figure 7. Auger elemental mapping of the wear track.
SEM image of the wear track (a) and the corresponding Auger elemental maps of carbon (b), molybdenum (c), and sulfur (d) from the wear track and areas nearby.

(a)

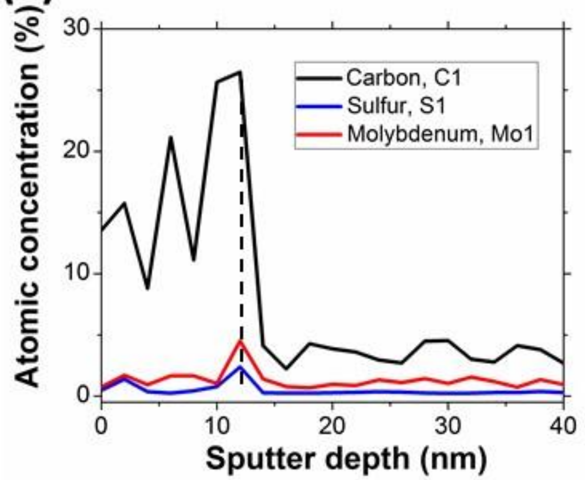

(b)

Elements sampled by Auger Electron Spectroscopy	Atomic concentration (in atomic %)	
	Inside wear track	Outside wear track
Carbon, C1	15.0	47.2
Oxygen, O1	83.9	49.3
Sulfur, S1	0.2	1.2
Molybdenum, Mo1	0.9	2.3

Supplementary Figure 8. Auger depth profile of the wear track

(a) Auger depth profile of the wear track for carbon, sulfur and molybdenum (dotted line indicates an approximate thickness of the tribolayer) and (b) corresponding atomic percentage of various elements probed from inside and outside of the wear track.

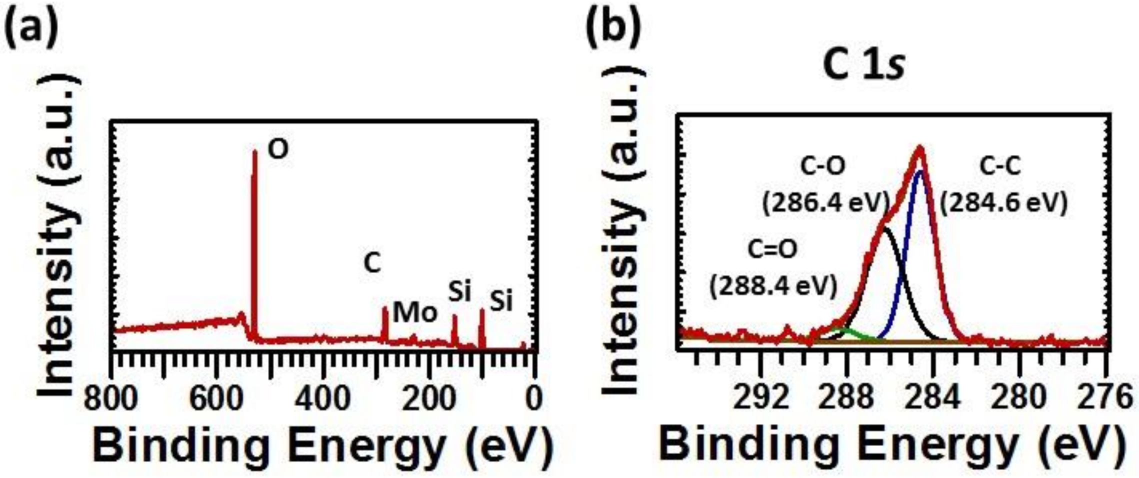

Supplementary Figure 9. XPS survey and detailed carbon C1*s* scan.

(a) XPS survey scan and (b) high resolution scan at C1*s* edge indicating presence of oxygen, chemically bonded to the carbon inside the wear track.

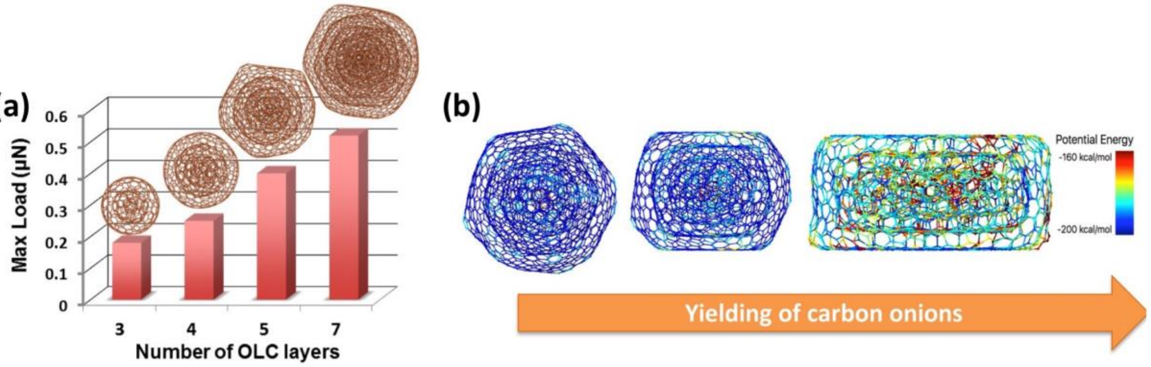

Supplementary Figure 10. Load-bearing properties of layered carbon onions .
(a) Dependence of fracture loads on number of layers in the carbon onions, indicating increased load-bearing stability of OLC with increase in OLC layers, (b) Atomic snapshots during the compression of the OLC are shown for the structure with 7 layers (the atoms are colored by their potential energy). The initially round shape of OLC is subjected to deformation and the limit of the load-bearing capability is determined based on bond breaking at the center of the structure.

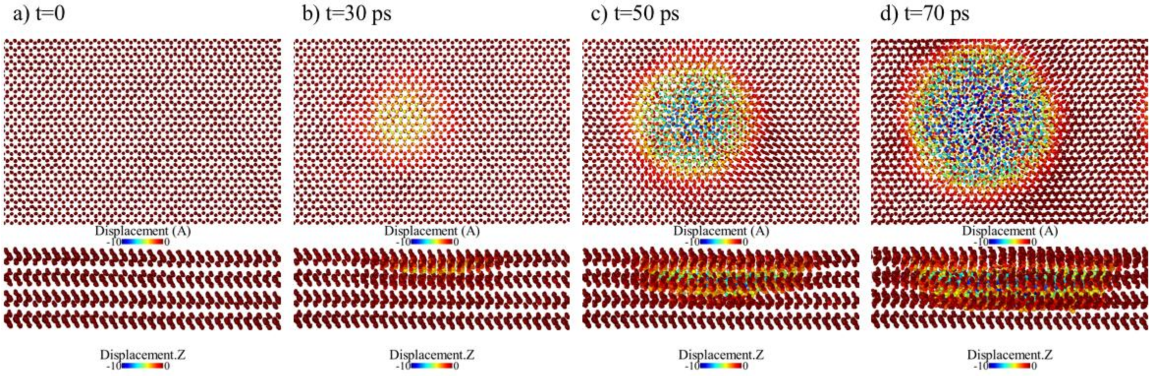

Supplementary Figure 11. Physical rupture of MoS_2 sheets under high contact pressure. Snapshots above depict the temporal evolution and reveal the disorder in the MoS_2 sheet after the pressure is applied through an indenter.

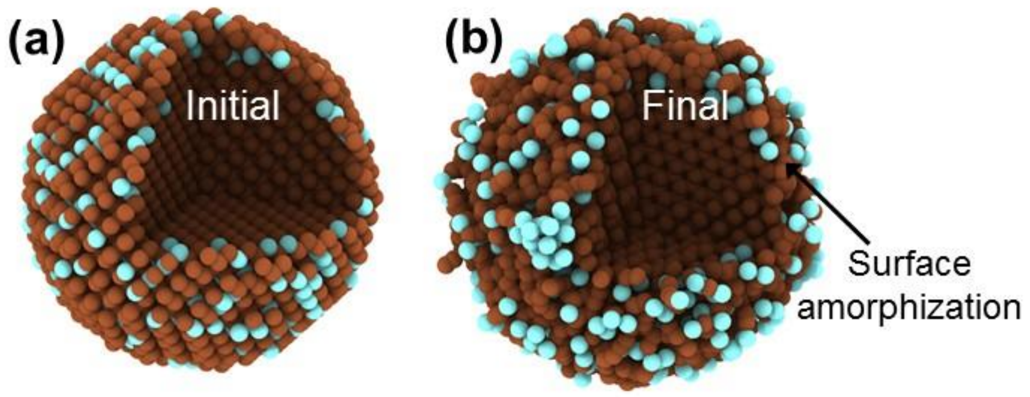

Supplementary Figure 12. Graphitization of nanodiamond in presence of molybdenum.
(a) Mo reacts with C atoms on the surface of diamond particle forming carbide, and induces amorphization at the surface at 2000 K; (b) within MD timescales, the amorphization front does not traverse the whole particle and remains restricted to the surface. Atomic snapshots are shown here at (a) t = 0 and (b) t = 1 ns. The C atoms are depicted as brown spheres, while Mo atoms are represented by cyan spheres. The formation of molybdenum carbide is thermodynamically feasible under high local temperatures (near asperities) typically observed under dry sliding conditions [1].

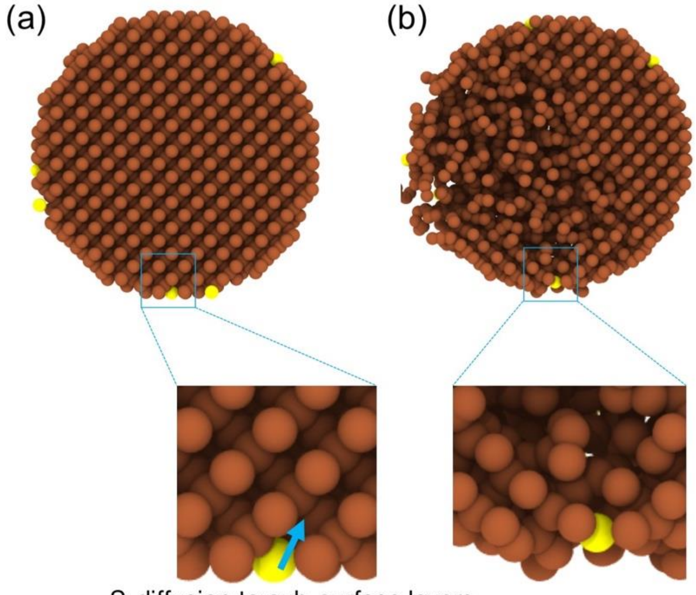

Supplementary Figure 13. Movement of S atoms from the surface of the nanodiamond towards the core. (a) Initial nanodiamond with S atom on the surface and (b) nanodiamond after S-induced lattice disordering. The corresponding insets showing the disordering process. We show here a slice through the sphere to observe the evolution of the surface, as well as the bulk of the nanodiamond sphere. Zoomed in insets show the inward movement of a representative surface S atom (indicated by blue arrow). This inward S-diffusion is accompanied by amorphization of the diamond lattice. The C and S atoms are depicted as brown and yellow spheres respectively.

Supplementary Table 1. Strain and defect energies of various dopants in a diamond lattice calculated using DFT.

**Dopant**	**Substitution**		**Interstitial**	
	**Vol. Strain (%)**	**Defect (eV)**	**Vol. Strain(%)**	**Defect (eV)**
S	2.37	10.94	6.04	30.42
B	0.47	1.17	2.95	18.01
O	1.15	3.12	3.26	15.94
N	0.29	0.93	3.25	17.12

Supplementary Note 1. Importance of the nanodiamond + MoS_2 combination

To further elaborate the importance of the nanodiamond + MoS_2 combination for successful OLC formation and thus near-zero COF, we performed a test for pure MoS_2 sliding against the DLC surface (Supplementary Figure 3). In this case limitation of free carbon suppresses formation of OLC and results in the high COF.

Also, to demonstrate the uniqueness of forming large OLC structures for demonstrating ultra-low friction, we have performed a tribo-test with small 5-7 nm OLCs (Supplementary Figure 4a). The tests for bare small OLC (Supplementary Figures 4b and c) as well as for small OLCs combined with molybdenum disulfide (Supplementary Figures 4d and e)

revealed comparably low friction, although not in the superlubricity regime. These results demonstrate the necessity for forming larger structures *in-situ* to promote near zero friction. As demonstrated by MD simulations, the large OLC structures show higher stability. Mixing small OLC with MoS_2 is also not efficient for forming large stable onions due to limitations of available carbon source, or for forming MoS_2 scrolls due to the absence of dangling bonds in OLC to initiate the scrolling. Also, for the case of manually supplied OLC, the produced wear of the H-DLC ball is also larger than the wear for the superlubricity case described in the main manuscript.

Supplementary Note 2. Auger and XPS analysis.
Auger electron spectroscopy (AES) was used for elemental mapping during depth profiling of the wear track. X-ray photoelectron spectroscopy (XPS) was used for characterizing the chemical state of the wear track. Given the atomically thin nature of MoS_2 flakes, AES and XPS nicely compliment each other in terms of overcoming their sensitivity limitations thus enabling detection of elemental composition and surface chemistry on the very top surface and at the bottom of the wear track. The reference SEM image of the wear track and the corresponding Auger elemental maps of carbon, molybdenum, and sulfur from the wear track are shown in Supplementary Figure 7(a-d) respectively, indicating high concentration of carbon in the wear track with weak contribution from the molybdenum and sulfur in the sampling area. Considering the typical sampling depth in AES of about 5 nm the distribution of elements detected could be within this range. The Auger elemental depth profiling within the wear track and corresponding atomic percentage of various elements probed from inside and outside of the wear track are shown in the Supplementary Figure 8. The dotted line in Supplementary Figure 8(a) represents the approximate thickness of the tribolayer. It is interesting to see that molybdenum and sulfur are sitting at the bottom of the wear track in very small quantity which supports our earlier prediction and LDSPI data. Low signal of the sulfur inside the wear track indicates that most of the sulfur is consumed in the tribocatalytic activity when converting nanodiamonds into OLCs and only residual sulfur finally settles down at the bottom of the wear track. Another interesting observation is presence of high amount of oxygen within the wear track. We attribute this to the porous nature of the OLCs formed within the wear track which could easily adsorb oxygen. Oxygen adsorption is clear from the X-ray photoelectron spectroscopy (XPS) results in the Supplementary Figure 9 (a-b), showing high amount of oxygen on the surface in the survey scan and chemically bonded to carbon shown in the high resolution scan at C1*s* edge respectively. It is important to note that no sulfur was detected on the surface using XPS, which supports AES and LDSPI results indicating sulfur was mostly either at the sub-surface regions (below 5nm from top) or sitting at the bottom of the wear track.

References

[1] Massalski, T. B., Okamoto, H., Subramanian, P. & Kacprzak, L. Binary alloy phase diagrams. vol. 3. *ASM Int.*, *1990*, 1485 (1990).
