# Supplementary material for: Operando tribochemical formation of onion-like-carbon leads to macroscale superlubricity
Source: Nat Commun. 2018 Mar 21;9:1164. doi: 10.1038/s41467-018-03549-6 (PMC5862981; doi:10.1038/s41467-018-03549-6)
Supplement: Supplementary file 2 — Description of Additional Supplementary Information(PDF 50 kb) [file 41467_2018_3549_MOESM2_ESM.pdf]

Description of Additional Supplementary Files

File Name: Supplementary Movie 1

Description: RMD movie showing the formation of ordered OLC from amorphous carbon matrix containing uniformly dispersed S atoms (15% S concentration) during cooling from 1000 K to 300 K over 2 ns. Initially, the S atoms (shown in yellow) segregate resulting in C-rich domains (shown in brown). Subsequently, the C atoms within these domains organize into well-ordered nanostructures with onion-like morphology. In this movie, we present the magnified view of a representative C-rich region (red circle), where graphitic structures nucleate, and grow into well-ordered OLC with trace amounts of sulfur. The OLC consists of multiple concentric spheres of C, each possessing graphitic order, held together by dispersive forces.
